# Real‐Time Stress Visualization of Hydrogels Enabled by Supramolecularly Switched Stretch‐Induced Phase Separation

**DOI:** 10.1002/advs.76110

**Published:** 2026-06-18

**Authors:** Sooyeon Noh, Akihide Sugawara, Naoaki Ishihara, Takashi Konishi, Yuki Ueda, Ryuhei Motokawa, Yoshinori Takashima, Hiroshi Uyama

**Affiliations:** ^1^ Department of Applied Chemistry Graduate School of Engineering The University of Osaka Suita Japan; ^2^ Graduate School of Human and Environmental Studies Kyoto University Kyoto Japan; ^3^ Department of Physics Ritsumeikan University Kusatsu Japan; ^4^ Materials Sciences Research Center Japan Atomic Energy Agency Tokai Japan; ^5^ Department of Macromolecular Science Graduate School of Science Institute for Open and Transdisciplinary Research Initiatives (OTRI) Forefront Research Center The University of Osaka Toyonaka Japan

**Keywords:** adamantane, materials science, mechanotransduction, opacity, polymer, supramolecular chemistry, visualization

## Abstract

The visualization of mechanical stress in soft materials is highly desirable; however, real‐time optical readouts using conventional sensing approaches are problematic because mechanophore‐based systems typically require strong threshold‐type activation with slow recovery. Herein, we report supramolecular hydrogels that enable the continuous and reversible visualization of mechanical stress in real time via stretch‐induced phase separation. Supramolecular switching mechanotransduction (SSM) has been proposed as the key mechanism. Mechanical stimuli are transduced into a distinct network state transition through host–guest complexes between β‐cyclodextrin and adamantane as supramolecular switches. In this design, guest‐functionalized polymers undergo on–off transition between the hydrated and dehydrated states via host–guest complexation and decomplexation. Responsive polymers, incorporated into the hydrogel network via supramolecular bonds, function as reversible cross‐links and switches. Upon stretching, the hydrogels macroscopically transition from transparent to opaque owing to the dehydration‐induced heterogeneity within the responsive domains. The linear and reversible changes in the opacity with applied stress—attributable to sacrificial and reversible supramolecular switching—enable the visualization of stress distributions. This design principle offers a platform for spatiotemporally resolved mapping of the mechanical states in hydrogels with an intuitive, instrument‐free readout, and lays the foundation for monitoring, timely intervention, and safer operation of soft‐material systems.

## Graphical abstract

Supramolecular hydrogels enable real‐time visualization of mechanical stress through supramolecular switching mechanotransduction. Adamantane‐modified cellulose functions as a responsive domain that undergoes an on–off transition between hydrated and dehydrated states via reversible β‐cyclodextrin–adamantane complexation and decomplexation. Mechanical deformation activates this switching process, inducing reversible opacity changes that enable continuous mapping of stress distributions.

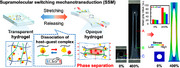

## 1 Introduction

**FIGURE 1:**
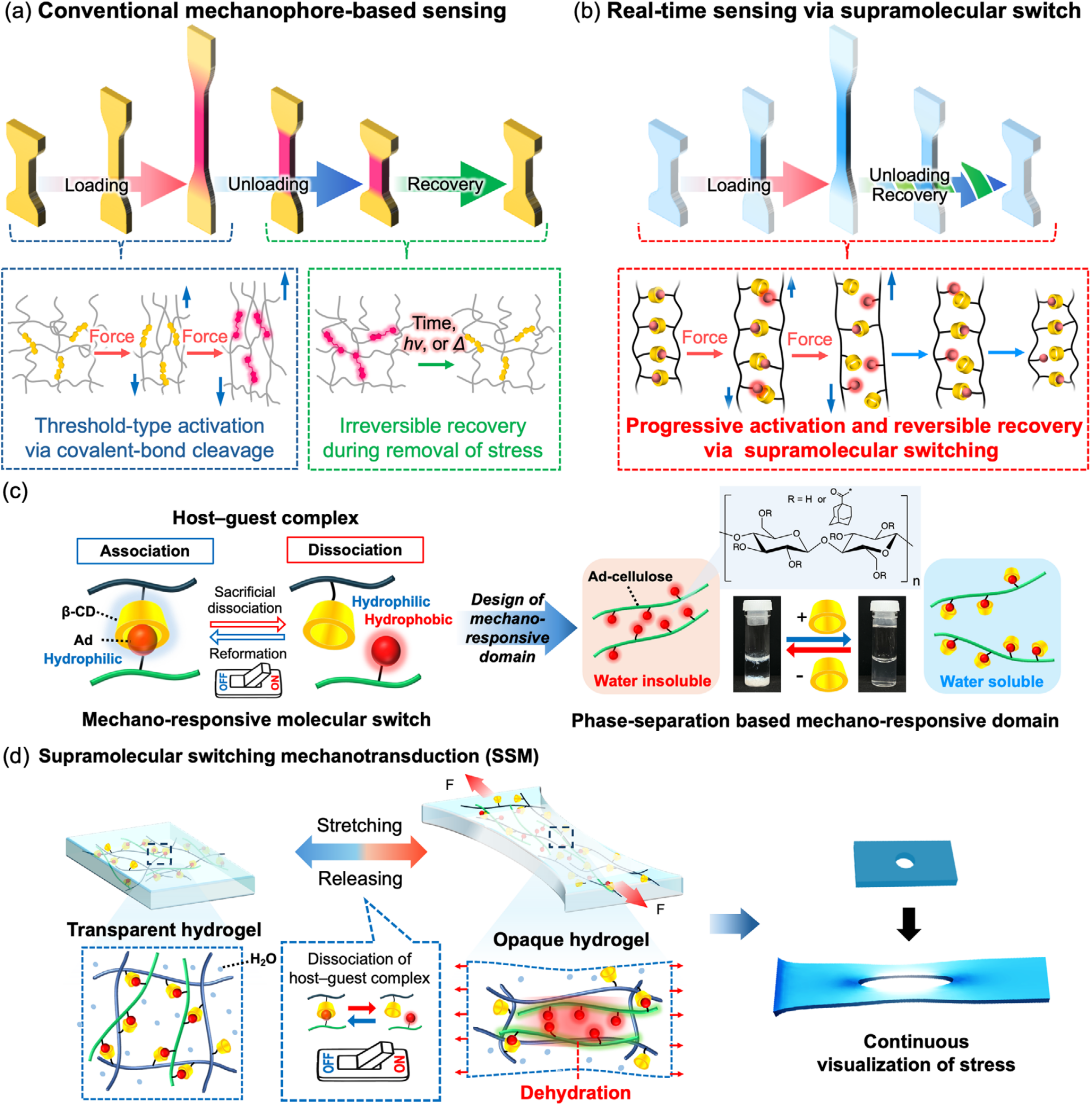
(a,b) Schematic illustrations of the design of conventional mechanophore‐based sensing and real‐time sensing via supramolecular switching. Schematic representations of the (c) design of the mechano‐responsive domain based on dehydration, and (d) reversible opacity changes in the hydrogel upon stretching and releasing, enabled by the host–guest complex, which serves as a molecular switch via supramolecular switching mechanotransduction (SSM) to allow visualization of stress within the hydrogel.

Mechanoresponsive materials that visually signal mechanical stress via macroscopic changes, such as changes in color or luminescence, have gained increasing attention for applications in damage detection, force mapping, and optical sensing [1, 2, 3, 4]. Their ability to provide a direct and intuitive means of reading mechanical input is advantageous in systems that require real‐time monitoring and noninvasive feedback. This capability is critical for hydrogels, which are soft, wet materials with biomimetic properties, yet their inherently low mechanical strength renders stress sensing highly desirable [5]. Various strategies have been explored to induce mechanoresponsive optical changes in hydrogels. One pioneering and widely explored strategy involves embedding mechanophores in the polymer matrices, where the applied mechanical force induces molecular structural or chemical transformations that result in macroscopic optical responses such as color changes or luminescence [6, 7, 8, 9, 10]. Another approach employs photonic crystals, in which periodically ordered structures, including colloidal or polymeric lattices, produce strain‐dependent structural color shifts [11, 12, 13, 14, 15]. Although these approaches exhibit clear mechanochromic functionality, photonic crystal systems are prone to experiencing color changes that depend on the viewing angle owing to Bragg diffraction [16, 17], which limits their applicability in quantitative stress mapping. Moreover, various mechanophore‐based systems rely on the scission of covalent bonds within the mechanophore, which results in irreversible responses (Figure 1a) [18, 19, 20]. Mechanophore activation typically requires substantial extension of the polymer chain, which occasionally approaches material fracture, and leads to a threshold‐type response rather than a continuous response to mechanical stress [21, 22]. This issue is highly problematic in soft materials, because their uniformly distributed stress limits reversible and real‐time sensing [23]. To overcome these limitations, mechanoresponsive systems require reversible and fatigue‐resistant molecular motifs that can repeatedly respond to stress without permanent structural damage.

Supramolecular bonds, particularly when used as cross‐links in hydrogels, act as sacrificial bonds that can dissociate under mild mechanical stress and reversibly reassociate, thereby mitigating stress concentration and preventing irreversible network fracture [24, 25, 26]. Among these supramolecular bonds, host–guest complexes formed between β‐cyclodextrin (β‐CD) and adamantane (Ad) serve as strong yet reversible cross‐links that impart high toughness and self‐healing properties to hydrogels [27, 28, 29, 30, 31, 32, 33, 34]. Furthermore, the heteromolecular nature of the β‐CD–Ad pair underscores its unique ability to function as a molecular switch in response to mechanical stimuli [35, 36]. Composed of a hydrophilic host (β‐CD) and a hydrophobic guest (Ad), this complex translates mechanical inputs into pronounced changes in the local hydration environment. Specifically, upon complexation, the hydrophobic Ad moiety is fully encapsulated within the cavity of β‐CD, thus rendering the cross‐linking site hydrophilic [37, 38, 39]. However, mechanical dissociation releases Ad, which markedly increases the local hydrophobicity. Consequently, the force‐induced association and dissociation of the β‐CD–Ad pair produces an effective on–off transition between the hydrophilic and hydrophobic states. Notably, these substantial environmental changes are triggered by molecular recognition–driven Ångström‐scale displacements [40, 41]. Such a highly localized switching mechanism is difficult to realize using long‐range interactions such as electrostatic forces [42]. Accordingly, the β‐CD–Ad molecular switch affords sharp and sensitive mechanical responses that allow the mechanical stress to be continuously and reversibly sensed (Figure 1b). However, in conventional host–guest cross‐linked hydrogels, mechanical stress does not directly produce macroscopic optical responses because complex dissociation alone is insufficient to generate a large optical contrast within the highly hydrated and relatively homogeneous gel network. The realization of supramolecular mechanoswitches demands network designs that convert molecular‐scale host–guest dissociation into localized heterogeneous structural changes coupled to optical outputs. This concept and its associated mechanism are collectively termed supramolecular switching mechanotransduction (SSM).

In this paper, we report mechanoresponsive hydrogels that exhibit continuous and reversible macroscopic optical changes based on SSM, in which mechanical deformation induces local dehydration and transient heterogeneous states within the hydrogel network. Stress‐induced dehydration within the hydrogel network causes a visible transition from a transparent to an opaque state, which enables direct and instrument‐free visualization of stress under visible light. To realize SSM based on this mechanism, we designed guest‐functionalized polymers as phase‐separation domains, where association and dissociation with host molecules are accompanied by reversible hydration and dehydration of the polymer chains in an on–off manner (Figure 1c). The incorporation of these domains into hydrogel networks via supramolecular crosslinking enables mechanical stretching to trigger host–guest molecular switching, which, in turn, increases the local hydrophobicity around the guest moieties, results in subsequent dehydration‐induced heterogeneity, and causes localized phase separation of the domains. This heterogeneity gives rise to a clear on–off transition in the network state, which constitutes a key step in SSM. Moreover, the load‐dependent dynamics of the supramolecular switches enable hydrogels to respond continuously and reversibly to modest mechanical stresses. By integrating supramolecular switching with the formation of a stress‐induced heterogeneous state, this study demonstrates a general design principle for continuous and reversible mechanoresponsive soft materials, which allows the visualization of mechanical stress and provides a basis for stress mapping in hydrogels (Figure 1d).

## 2 Results and Discussion

### 2.1 Design of Supramolecular Hydrogels for Stretch‐Induced Phase Separation

Continuous and reversible mechanoresponsive behavior requires amplification of the mechanical stimuli beyond molecular‐scale changes to network‐level transitions that are observable under visible light. As a design strategy, stretch‐induced dehydration was employed as the basis for mechano‐responsiveness. In this regard, the β‐CD–Ad complex, which served both as a molecular switch and as a supramolecular cross‐linker, acted as the mechanism by which mechanical stimuli are converted into transformations of the phase‐separation domains. On this basis, phase‐separation domains were designed such that they undergo SSM, function within a hydrogel network, and exhibit highly sensitive on−off switching between the hydrated and dehydrated states in response to association and dissociation with host molecules.

Accordingly, cellulose was selected as the backbone of the phase‐separation domains because, despite having a hydroxyl‐rich hydrophilic structure, it remains insoluble in water owing to strong intermolecular hydrogen bonding among the hydroxyl groups. The introduction of a controlled amount of hydrophobic Ad groups is known to maintain the water‐insoluble state of Ad‐cellulose via both the hydrophobic association of the Ad groups and hydrogen bonding among the hydroxyl groups [43]. In contrast, inclusion complexation with β‐CD suppresses the hydrophobic association of these Ad groups and weakens the intermolecular interactions, thereby enabling solubility switching in water. With this in mind, Ad‐modified cellulose (Ad‐cellulose) with controlled degrees of substitution (DS) was synthesized and characterized (Figures S1a,b and S2) to achieve reversible solubility switching depending on the presence or absence of β‐CD molecules. Ad‐cellulose with a DS of approximately 0.2 was found to be insoluble in water in the absence of host molecules, whereas association with 6‐acrylamido‐β‐CD (β‐CDAAm) induced hydration and clear solubility switching of Ad‐cellulose (Figure 1c). This behavior was further supported by the ^1^H NMR spectra, with the appearance of peaks at 1.5–2.0 ppm corresponding to the Ad groups (Figure S3). These results demonstrate that the formation of the host–guest complex between the Ad groups and β‐CD derivatives enhances the overall hydrophilicity of Ad‐cellulose to facilitate its dissolution in water. Thus, based on the design of the phase‐separation domains, a guest‐modified polymer exhibiting clear hydration switching in response to host complexation was successfully realized.

**FIGURE 2:**
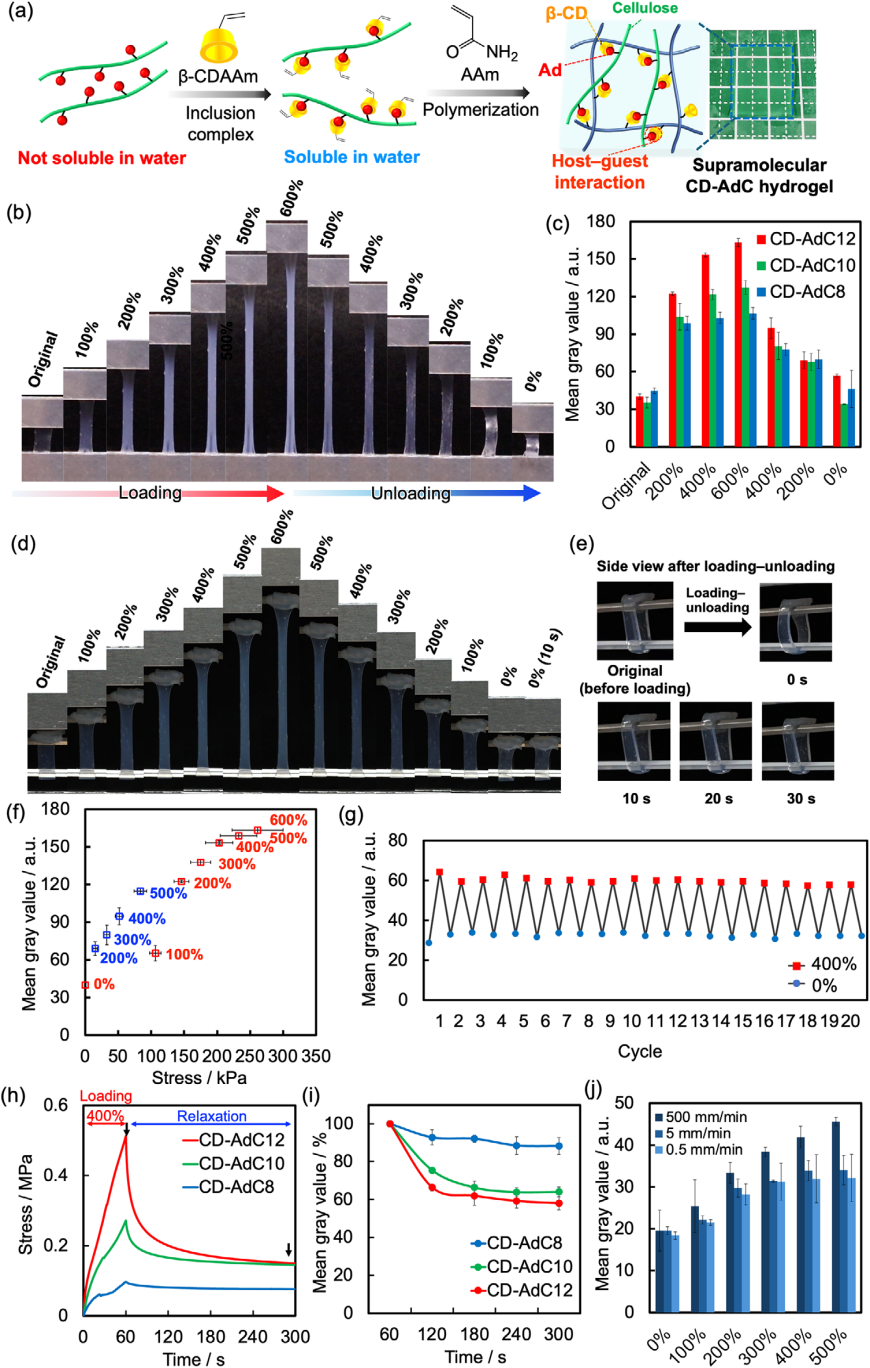
Stretch‐induced mechano‐responsive behavior of CD−AdC hydrogels. (a) Schematic illustration of the preparation of supramolecular CD−AdC gels. (b) Photographs of the CD−AdC12 gel during the repeated loading‐unloading test. (c) Corresponding mean gray values of the CD−AdC gels. (d) Photographs of the ring‐shaped CD–AdC8 gel during cyclic loading–unloading. (e) Side‐view photographs of the ring‐shaped CD–AdC8 gel during the recovery process for 30 s after unloading. (f) Correlation between applied stress and mean gray value at various tensile strains during loading–unloading tests of the CD–AdC12 gel. (g) Corresponding mean gray values of the ring‐shaped CD−AdC8 gel during the cyclic test. The tests were conducted with a 3 s holding period at the maximum strain and a 10 s holding period after unloading. (h) Stress relaxation curves of CD−AdC gels during loading to 400% strain, followed by a 240 s hold at constant strain. (i) Corresponding percentage change in mean gray values, normalized to the value at the start of the stress relaxation (measured at 60 s). (j) Mean gray values corresponding to the photographs of CD−AdC8 gels stretched at different tensile speeds.

The mechano‐responsive hydrogel was prepared by copolymerizing acrylamide (AAm) and β‐CDAAm in the presence of Ad‐cellulose (Figure 2a). The hydrogels are denoted as CD–AdC or CD–AdC*x* gels, where *x* represents the weight percentage of Ad‐cellulose in the pre‐gel solution. The hydrogels thus formed a supramolecular cross‐linked network, in which Ad‐cellulose was incorporated as phase‐separation domains in a hydrated state through complexation with the β‐CD moieties in the matrix. The amount of β‐CDAAm was optimized to approximately 1.2 equivalents relative to the Ad groups in Ad‐cellulose to achieve both sufficient hydration of Ad‐cellulose and stable gel formation. The transparent appearance of the resulting hydrogels indicated the hydration of Ad‐cellulose within the network. The FTIR spectrum of the hydrogel exhibited the characteristic amide I and amide II absorption bands at approximately 1650 and 1540 cm^−1^, respectively, confirming the formation of the polyacrylamide‐based network (Figure S4). In addition, absorption bands attributable to the β‐CD molecules in the 1000–1150 cm^−1^ region indicated the presence of β‐CDAAm within the hydrogel network. Furthermore, ^1^H NMR analysis of the precursor solution before and after polymerization revealed that the vinyl proton signals at 5.5–6.0 ppm disappeared after polymerization, indicating that both monomers were nearly completely converted during copolymerization (Figure S5).

### 2.2 Stretch‐Induced Mechano‐Responsive Behavior of Supramolecular Hydrogels

The mechano‐responsive behavior of the CD−AdC gels was investigated using loading‐unloading tensile tests. The changes in the macroscopic appearance of the gels were recorded under visible light irradiation. The initially transparent CD−AdC gels gradually became opaque upon stretching and returned to their original transparent state once the applied stress was discontinued (Figure 2b; Figure S6 and Movie S1). The corresponding loading–unloading stress–strain curves of the CD–AdC gels are shown in Figure S7. The mean gray value was calculated from the images to quantify the degree of opacity of the different strains. Figure 2c shows the mean gray value of the CD−AdC gels during loading and unloading cycles up to 600% strain. During loading, the mean gray value gradually increased to reach its highest value at 600% strain, whereas during unloading, a decreasing trend was observed. These changes in opacity are attributed to stretch‐induced dehydration, which leads to structural heterogeneity and localized phase separation of Ad‐cellulose within the network, triggered by the dissociation of host–guest complexes as a molecular switch. As the strain increased, progressive dissociation of the host–guest complexes promoted the dehydration of Ad‐cellulose, whereupon the gel took on a whiter appearance. Upon unloading, reassociation between the β‐CD and Ad moieties resulted in rehydration of Ad‐cellulose, allowing it to recover its initial hydrated state and undergo reversible mechano‐responsive behavior. This behavior differs from that of conventional CD–Ad hydrogels, which are devoid of designed phase‐separation domains, and which typically do not exhibit visible and reversible turbidity changes under deformation [29]. The CD–AdC12 gel had the highest mean gray value among the examined gels, indicating that a higher concentration of phase‐separation domains leads to a more pronounced increase in turbidity under stretching.

To further evaluate the recovery behavior after large‐scale deformation, loading–unloading tensile tests were additionally performed using ring‐shaped CD–AdC8 gels to minimize the influence of mechanical clamping during substantial deformation and enable a more accurate evaluation of the intrinsic recovery behavior (Figure 2d and Movie S2). The corresponding loading–unloading stress–strain curve of the ring‐shaped CD–AdC8 gel is shown in Figure S8. The ring‐shaped gel became opaque under tensile strain of 600% and recovered its transparent appearance after unloading. In addition, side‐view photographs revealed that the original shape and appearance of the gel were restored within 30 s of unloading (Figure 2e), further supporting the reversible nature of the mechano‐responsive behavior.

The continuity of the mechano‐responsive behavior was evaluated by analyzing the correlation between the mean gray value and applied stress (Figure 2f). Interestingly, the relationship between the applied stress and mean gray value was approximately linear for the CD–AdC12 gel over a range of tensile strains during both loading and unloading. This linear correlation, which demonstrates a nearly proportional response of the mean gray value to the mechanical stress, enables a semiquantitative evaluation of the relative stress changes and stress distribution during deformation.

The reproducibility of the mechanoresponsive behavior was investigated by conducting cyclic loading–unloading tests up to 400% strain. During these tests, the ring‐shaped CD–AdC8 gel reproducibly transitioned from the transparent to the opaque state, even after 20 cycles, demonstrating its consistent and repeatable mechano‐responsive behavior (Figure 2g and Figure S9a,b). Upon repeated stretching and release after the second cycle, the mean gray value decreased slightly, likely reflecting the accumulation of residual strain in the gel. These results demonstrate that CD–AdC gels undergo continuous, reversible, and repeatable changes in their opacity in response to mechanical forces via stretch‐induced dehydration driven by SSM.

The stress‐relaxation behavior of the CD−AdC gels was investigated to gain deeper insight into their time‐dependent mechano‐responsive behavior [44]. The hydrogels were stretched to a strain of 400% and held in the deformed state for 240 s (Figure 2h and Figure S10). Upon stretching, the stress increased sharply and then gradually relaxed to stabilize after the specified amount of strain was applied for a fixed period, thus exhibiting characteristic stress relaxation behavior. The extent of stress relaxation was strongly dependent on the cellulose content of the gels. In particular, gels with higher Ad‐cellulose concentrations exhibited greater stress decay during relaxation, whereas the relaxation response of CD–AdC8, which had the lowest Ad‐cellulose content, was suppressed. This suggests that a higher density of reversible supramolecular interactions enhances stress dissipation during relaxation. To facilitate comparison of the time‐dependent responses among the samples, the mean gray value during stress relaxation was normalized to the maximum value (non‐normalized data are shown in Figure S11). The normalized mean gray value responded in a distinctly time‐dependent manner coupled with mechanical relaxation behavior (Figure 2i). When the applied strain was released, the opacity decreased as a function of time until it reached a plateau, closely tracking the stress–relaxation behavior of the respective gels. The turbidity of CD−AdC12 decreased the most, whereas that of CD−AdC8 only decreased slightly during relaxation. This behavior is attributed to the reorganization of and stress redistribution within the internal network during stress relaxation, which partially lowers the localized stress concentration that accumulates during stretching. During this process, dissociated host–guest complexes are considered to partially reassociate, accompanied by the rehydration of the Ad‐cellulose domains. Consequently, the extent of dehydration gradually decreases during relaxation, accompanied by an observed reduction in turbidity. This behavior was particularly pronounced for CD−AdC12, which exhibited the largest stress relaxation response, thereby supporting the dynamic and reversible nature of the heterogeneous structure. These observations indicate that the phase separation domains and molecular switches incorporated into the network exhibit real‐time dynamic mechanoresponsive behavior through internal reorganization under applied tensile stress.

Next, the effect of the deformation rate on the mechano‐responsive behavior was examined. Tensile tests were conducted on the CD−AdC8 gel at strain rates of 0.5, 5, and 500 mm/min. The gel became noticeably more opaque at higher rates, whereas only a modest change in opacity was observed at lower rates (Figure 2j and Figure S12). This difference in behavior correlated with the internal stress observed under each stretching condition (Figure S13). The dependence on the strain rate of both the stress response and the associated optical behavior of the gels is attributed to the time‐dependent viscoelastic nature of the hydrogel network. At higher deformation rates, the relaxation of the network and reformation of supramolecular cross‐links are temporally constrained, which increases the internal stress. This, in turn, enhances dehydration, thereby increasing the turbidity. In contrast, under slower deformation, network rearrangement and reversible cross‐link reformation mitigate the accumulation of stress, suppress dehydration‐induced heterogeneity, and result in a smaller change in opacity. These results suggest that the opacity changes of CD–AdC hydrogels are closely correlated with the internal stress generated during deformation and reflect not only the magnitude of the mechanical stimulus but also its dynamic properties via the SSM mechanism.

### 2.3 Mechanical Properties of Supramolecular Hydrogels

**FIGURE 3:**
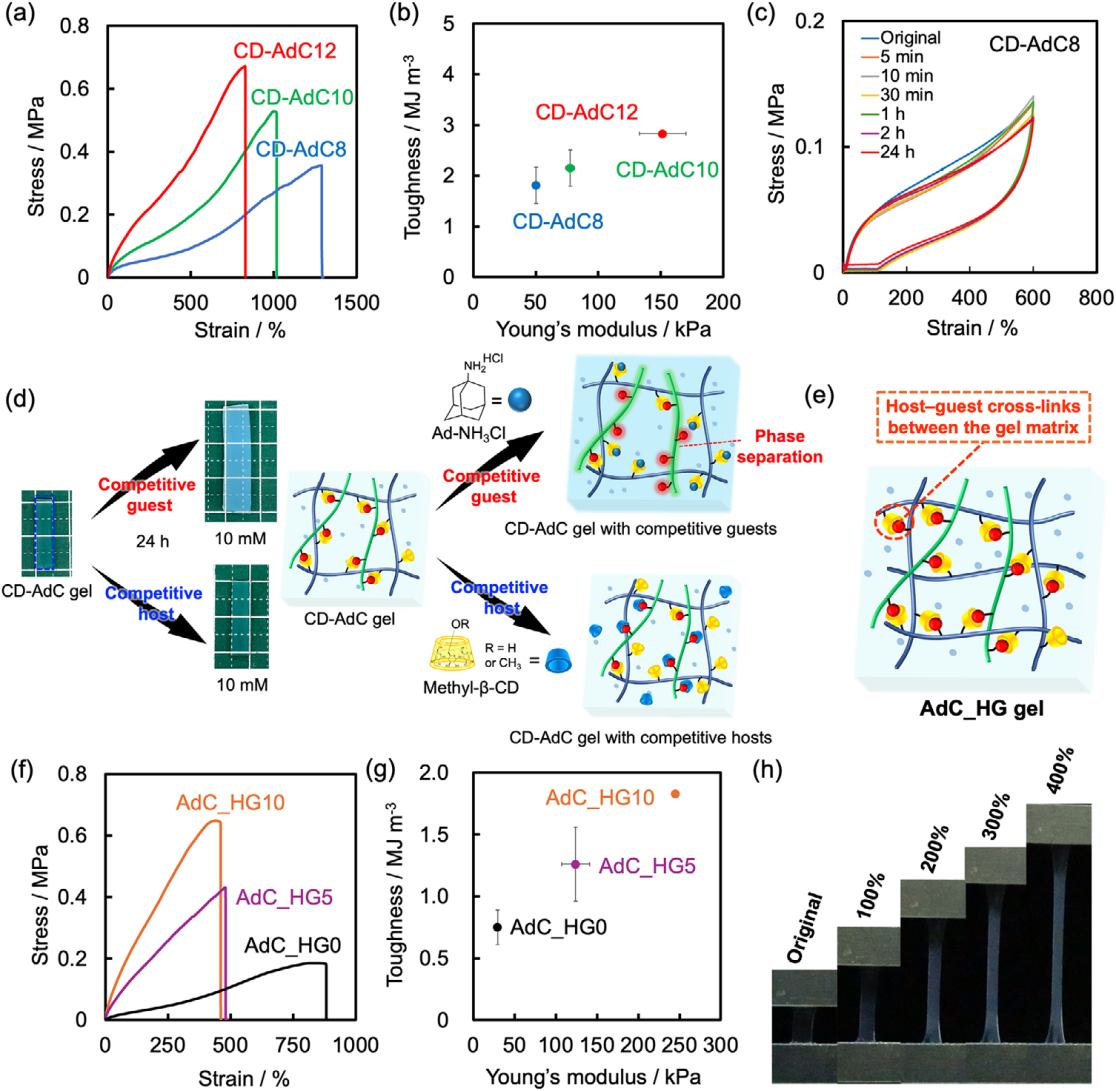
(a) Stress–strain curves and (b) relationship between Young's modulus and toughness of CD−AdC gels. (c) Stress–strain curves of CD−AdC8 hydrogels under cyclic tensile loading‐unloading tests, recorded by varying the waiting time from 5 min to 24 h. (d) Photographs of the CD−AdC8 gels immersed in aqueous solutions of competitive guest and host molecules, and a schematic illustration of gels immersed in aqueous solutions of competitive molecules. Phase separation of Ad‐cellulose was induced by competitive guests. Competitive hosts formed inclusion complexes with the Ad units on Ad‐cellulose. (e) Schematic illustration of the AdC_HG gel network. (f) Stress–strain curves and (g) relationship between Young's modulus and toughness of AdC_HG gels. (h) Photographs of the AdC_HG10 gel during tensile testing.

The mechanical properties of the CD−AdC gels were evaluated by tensile testing. The maximum stress of the CD−AdC gels increased significantly from 0.33 ± 0.04 MPa (CD−AdC8 gel) to 0.68 ± 0.02 MPa (CD−AdC12 gel) with increasing Ad‐cellulose concentration (Figure 3a). In addition, both the toughness and Young's modulus of the gels increased with increasing AdC content. Specifically, the toughness and Young's modulus of the CD−AdC12 gel were the highest, reaching 2.8 ± 0.03 MJ m^−3^ and 0.15 ± 0.02 MPa, respectively (Figure 3b). The Young's moduli of the CD–AdC gels were comparable to those of typical double‐network (∼0.1 MPa) and tetra‐PEG (∼0.05 MPa) hydrogels [45, 46]. These results indicate that increasing the Ad‐cellulose content reinforces the gel network through the combined effects of enhanced supramolecular cross‐linking and the intrinsic rigidity of the cellulose backbone.

The recoverability of the dissipated energy in the hydrogels was additionally evaluated by conducting tensile loading–unloading tests on the CD–AdC8 gel in water with different waiting times (Figure 3c). The applied strain was 600%, with waiting times ranging from 5 min to 24 h. The gel exhibited a pronounced hysteresis loop in the initial loading–unloading cycle, which is commonly observed in physically cross‐linked hydrogels. In subsequent loading–unloading cycles, the hysteresis loops that were recorded with various waiting times nearly overlapped with those of the initial cycle, indicating the effective recovery of the gel network. Furthermore, the shape of the hysteresis loop was essentially identical regardless of the waiting time up to 24 h, indicating that the host–guest cross‐links were re‐established within the first few minutes. These results highlight the dynamic and reversible nature of host–guest interactions in CD–AdC hydrogels, which enable the efficient recovery of the mechanical energy that was dissipated after deformation.

### 2.4 Dissociation of Host–Guest Cross‐Links by Competitive Molecules

The role of host–guest interactions in the mechano‐responsive behavior of the hydrogels was probed by immersing CD–AdC8 gels in aqueous solutions of competitive host or guest molecules for 24 h. 1‐Adamantanamine hydrochloride (Ad‐NH_3_Cl) and methyl‐β‐CD were selected as competitive guest and host molecules, respectively (Figure 3d). The addition of these competitive molecules induces the dissociation of supramolecular cross‐links instead of using mechanical stimuli. The marked reduction in the mechanical strength of the gels indicated the inhibition of cross‐linking by these competitive molecules (Figure S14).

Furthermore, the opacity of the gel immersed in the Ad‐NH_3_Cl solution increased noticeably, accompanied by swelling. This result indicates that complexation of the competitive guest with β‐CD moieties in the matrix induces the decomplexation of Ad moieties on Ad‐cellulose, which results in dehydration‐driven phase separation. In contrast, the gel immersed in methyl‐β‐CD solutions remained transparent, indicating that, although the host–guest cross‐linkers dissociated, Ad‐cellulose formed a complex with the competitive β‐CD and remained hydrated within the network without phase separation. These results strongly support the notion that the mechanoresponsive behavior originates from the phase separation of Ad‐cellulose induced by the dissociation of the host–guest complexes.

To further validate the role of the reversible host–guest reassociation in the recovery of the gel after deformation, an additional reverse validation experiment was performed using a competitive guest molecule under deformation. The CD–AdC8 gel, stretched to 400% strain, was immersed in an aqueous Ad‐NH_3_Cl solution for 1 h while maintaining its deformed state, followed by unloading. The gel treated with the competing guest remained opaque after unloading and did not fully recover its original length (Movie S3). This result indicates that competitive guest molecules complexed with β‐CD suppress reassociation between the β‐CD and Ad moieties on Ad‐cellulose after unloading, thereby inhibiting rehydration of Ad‐cellulose and recovery of the gel network. These findings provide further support for the proposed mechanism, according to which dissociation of the host–guest complexes induces phase separation of Ad‐cellulose during deformation, whereas reassociation after unloading promotes rehydration of Ad‐cellulose and recovery of the gel.

### 2.5 Controlling the Mechanical Properties of the Mechano‐Responsive Hydrogels via Incorporation of a Host–Guest Cross‐Linker

In a further experiment to generalize the mechanoresponsive design, *N, N*‐dimethylacrylamide (DMAAm) was used as the matrix monomer instead of AAm to construct an alternative network. In addition, a host–guest (HG) cross‐linker, based on β‐CDAAm and *N*‐adamantyl acrylamide (Ad‐AAm), was incorporated to reinforce the network structure (AdC_HG gels) even more (Figure 3e). The stress–strain curves demonstrate a clear enhancement of the mechanical strength with increasing HG crosslinker content (Figure 3f). The Young's modulus increased from 30 ± 2 kPa (AdC_HG0) to 0.25 ± 0.00 MPa (AdC_HG10), accompanied by a substantial improvement in toughness (Figure 3g). This enhancement indicated that the addition of the HG cross‐linker increased the effective cross‐linking density of the network, thereby improving the mechanical strength of the hydrogels. Notably, the AdC_HG gels maintained pronounced stretch‐induced mechanoresponsive behavior even after the incorporation of HG cross‐linking in the matrix polymer (Figure 3h). Despite the intrinsic turbidity of the AdC_HG0 gel prior to deformation, which limited clear visualization of the optical changes caused by stretching, the opacity of both AdC_HG5 and AdC_HG10 changed distinctly during the cyclic loading–unloading tensile tests (Figure S15). These results demonstrate that the mechano‐responsive behavior is not limited to the AAm‐based network but can be extended to other polymer matrices. Moreover, HG cross‐linking served to mechanically reinforce the gel while preserving the stretch‐induced dehydration of Ad‐cellulose, underscoring the versatility of the supramolecular design strategy.

### 2.6 Characterization of Network Structures

**FIGURE 4:**
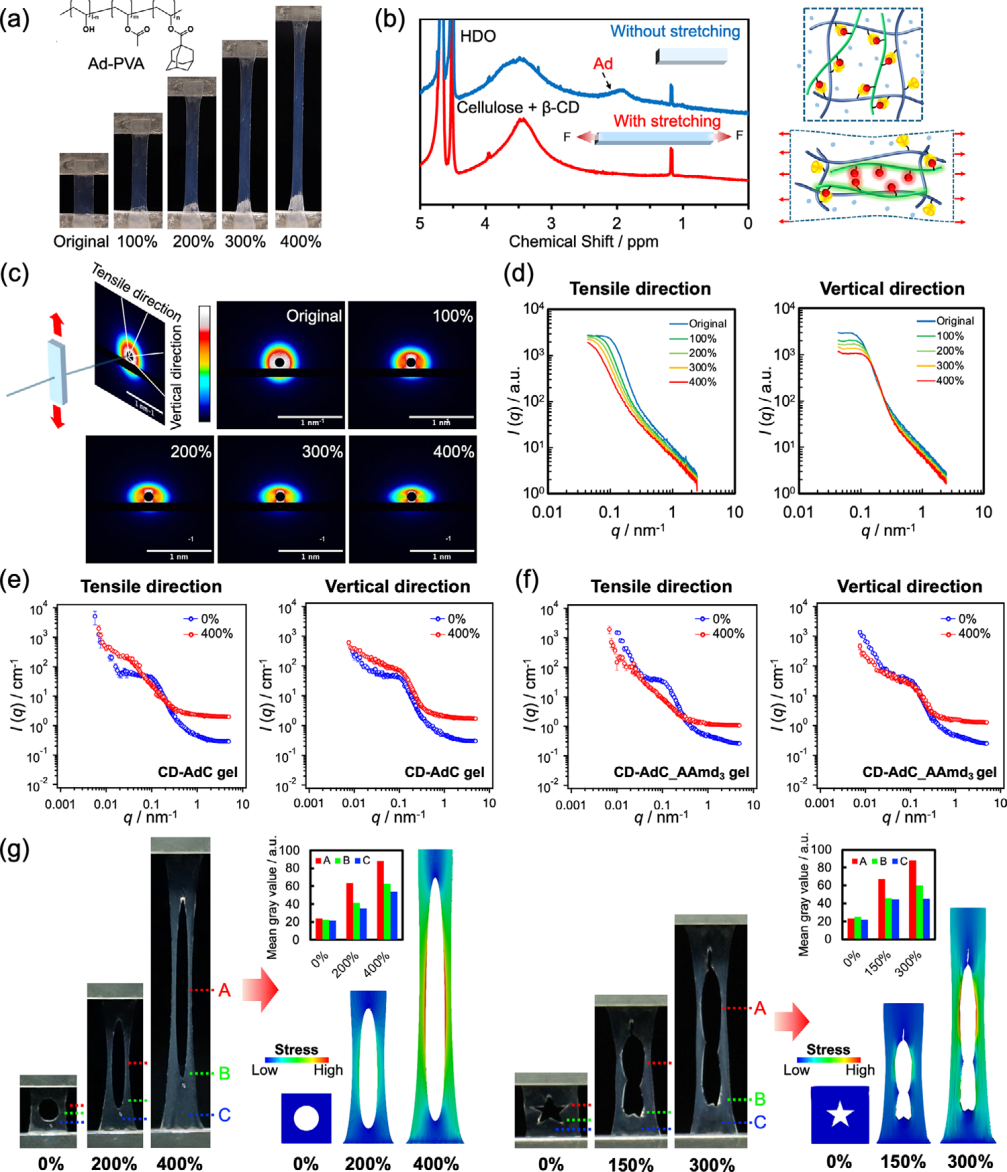
(a) Mechano‐responsive behavior of Ad‐PVA hydrogel. (b) ^1^H NMR spectra of CD–AdC8_AAmd_3_ gels in D_2_O with and without 300% stretching. (c) 2D SAXS profiles of CD–AdC gels during stretching. For both SAXS and SANS, sector‐averaged profiles were obtained along the tensile and vertical directions using angular sectors defined relative to the stretching direction of the gel. (d) 1D SAXS profiles of CD–AdC gels during stretching. 1D SANS profiles of (e) CD−AdC and (f) CD–AdC_AAmd_3_ gels along the tensile and vertical directions. (g) Photographs of CD–AdC hydrogels with circular and star‐shaped holes under tensile deformation (initial chuck length: 1 cm) and the corresponding simulated stress distribution results (Insets: mean gray values of the selected regions (A–C)).

As discussed in the previous sections, the solubility switching of Ad‐cellulose resulting from reversible complexation with and dissociation from β‐CD serves as the primary driving force for stretch‐induced dehydration of the gel network. Importantly, this design strategy is not limited to cellulose backbones. By utilizing poly(vinyl alcohol) (PVA), which possesses abundant hydroxyl groups capable of forming intermolecular hydrogen bonds and has limited solubility in cold water, as the backbone, solubility switching was realized in Ad‐functionalized PVA (Ad‐PVA). Hydrogels incorporating Ad‐PVA as a phase‐separation domain exhibited pronounced mechanoresponsive behavior upon stretching, demonstrating the versatility of our supramolecular design (Figure 4a).

The structural changes associated with the mechanoresponsive behavior of the hydrogels incorporating Ad‐cellulose as a phase‐separation domain were additionally characterized by recording ^1^H NMR measurements in D_2_O to evaluate the solvation state of the gel network (Figure 4b). To this end, CD−AdC8_AAmd_3_ gels were prepared using AAm‐d_3_ instead of AAm to enable the Ad‐cellulose signals to be selectively observed by eliminating the peaks derived from the AAm‐based main chains. A gel sample under constant strain of 300%, fixed to a rigid rod, was placed in an NMR tube for ^1^H NMR measurements (Figure S16). Unstretched gel samples were measured in the same manner. The spectrum of the unstretched gel displayed distinguishable signals assigned to the Ad moieties (at approximately 2 ppm) and to the cellulose and β‐CD moieties (3–4  ppm). In contrast, the signal intensity of the Ad peak of the stretched gel was markedly lower. Considering the heterogeneous and mechanically constrained state of the stretched gel, the attenuated Ad signals may reflect the restricted molecular mobility and structural heterogeneity in the stretched state [47]. These results suggested the development of structural heterogeneity within the gel network during stretching. Additionally, as a control experiment, conventional host–guest cross‐linked hydrogels (PAAmd3‐HG) were prepared using AAm‐d3 as the main chain component with HG as the cross‐linker, and their ^1^H NMR spectra were measured in both the unstretched and stretched states (Figure S17). In this case, two broad peaks attributable to the β‐CD and Ad moieties were still observed even in the stretched state, although the signals were broadened considerably upon stretching. These results suggest that the β‐CD and Ad moieties introduced into the polyacrylamide chains remained hydrated upon stretching. This behavior differed from that of the CD–AdC gel, for which the intensity of the Ad‐related peaks in the high‐field region (1.7–2.1 ppm) decreased considerably in the stretched state. These results suggest that the NMR detectability of the Ad‐cellulose component was reduced in the stretched state, possibly for reasons associated with local dehydration and structural heterogeneity. Based on the experimental results, the whitening behavior was attributed to the formation of a transient heterogeneous state induced by stress‐triggered local dehydration within the gel network. The resulting whitening behavior is fundamentally different from the conventional craze‐induced stress whitening in rigid plastics, where irreversible voids and crack‐like damage structures are typically generated during deformation [48]. In contrast, the gels exhibited reversible transparency changes upon unloading without macroscopic fractures or permanent structural damage. Moreover, SEM observations after stretching did not reveal clear voids, crack‐like structures, or brittle fracture features associated with the crazing behavior (Figure S18). These findings indicate that the whitening behavior primarily originates from local dehydration and transient heterogeneity, rather than irreversible damage. On the other hand, the stress–strain curves revealed strain‐hardening behavior at high strains, suggesting network orientation and densification during deformation. However, the uniform densification of a highly hydrated gel network alone is unlikely to generate a sufficiently large scattering contrast to produce the pronounced whitening observed in the present system. Rather, the NMR results and reversible transparency changes support stress‐induced local dehydration and the formation of local heterogeneous regions within the gel network through the dissociation of the host–guest complexes.

Similar reversible dehydration and rehydration processes have been widely discussed in thermoresponsive polymer systems such as poly(*N*‐isopropylacrylamide) near the lower critical solution temperature (LCST), where transient heterogeneous states and concentration fluctuations develop upon heating and disappear again upon cooling [49]. Similarly, in the present system, mechanical stretching was considered to transiently stabilize the locally dehydrated heterogeneous regions within the gel network. Therefore, the observed transparency change is interpreted not as an irreversible macroscopic phase separation but rather as a reversible stress‐induced heterogeneous state associated with local dehydration. A schematic of the energy landscape illustrating this interpretation is shown in Figure S19. Under stress‐free conditions, the hydrated homogeneous state is energetically favored, whereas stretching transiently stabilizes the locally dehydrated heterogeneous state. Upon unloading, the energy landscape returns to its original state, allowing rehydration and recovery of transparency.

The changes in the network structure upon stretching were also investigated by performing small‐angle X‐ray scattering (SAXS) and small‐angle neutron scattering (SANS) measurements. The SANS measurements were performed on the SANS‐J diffractometer of the JRR‐3 research reactor [50, 51]. Figure 4c shows the 2D SAXS profiles of the gels at various stretching ratios. The initially isotropic scattering pattern observed in the unstretched state of the gel gradually evolved into an elliptical shape with increasing strain. Further stretching to 400% resulted in the emergence of a distinct two‐lobe scattering pattern along the tensile direction. This pattern is similar to the abnormal butterfly pattern commonly observed for polymer gels owing to the spatial inhomogeneities in stretched anisotropic structures [52, 53, 54, 55]. 1D SAXS scattering profiles were obtained by sector averaging with a sector angle of ± 15° along the tensile direction and the direction vertical to stretching (Figure 4c,d). Along the tensile direction, the shoulder peak observed at *q* = 0.1–0.2 nm^−1^ appeared to shift toward lower *q* upon stretching. In contrast, along the vertical direction, this shoulder shifted toward higher *q* with stretching. These scattering features suggest the anisotropic emergence of sparse and dense regions along the stretching direction. Moreover, focusing on the change in the scattering slope around the higher *q* side of the shoulder region (*q* = 0.2 nm^−1^) before and after tensile deformation, the slope in the tensile direction became noticeably shallower, changing from approximately −4 to around −2 after stretching. In contrast, no significant change in the slope was observed in the vertical direction. These results indicate that the scattering in the aforementioned two directions originates from different structural features after deformation. Prior to stretching, the scattering was dominated by isotropic contributions from smooth interfaces following Porod's law. Subsequent tensile deformation reduces interfacial correlations in the vertical direction, while scattering from interfaces aligned along the tensile direction remained intact.

The 1D SANS profiles of the CD–AdC gel at 0% and 400% strains along the tensile and vertical (perpendicular) directions are presented in Figure 4e. In the unstretched state (0%), a shoulder was observed at *q* = 0.1–0.2 nm^−1^ in both directions, similar to the SAXS results. At 400% strain, the shoulder shifted to lower 𝑞 values along the tensile direction, whereas the shift along the vertical direction was not pronounced. These results are in good agreement with the SAXS observations described above, indicating that stretching induces anisotropic density variations within the network.

SANS measurements were also performed on the CD–AdC_AAmd_3_ gels prepared using AAm‐d_3_ instead of AAm (Figure 4f). In this case, scattering from the AAm component was suppressed and the contrast of the Ad‐cellulose component enhanced. In the unstretched state (0%), shoulder features similar to those of the CD–AdC gel were observed in both directions. Notably, at 400% strain, the low 𝑞 shoulder observed along the tensile direction in the CD–AdC gel disappeared, whereas the clear shoulder peak that remained in the vertical direction represented a marked difference between the CD–AdC_AAmd_3_ and CD–AdC gels. These results indicate that the Ad‐cellulose components become oriented along the tensile direction upon stretching, which lowers the scattering intensity along this direction while retaining relatively strong scattering in the perpendicular direction. In addition, the enhanced scattering intensity along the tensile direction observed in the SANS profiles of the CD–AdC gel originates from the AAm component. These results are also in good agreement with the SAXS observations described above. Overall, the contrast‐variation approach revealed that macroscopic stretching induces anisotropic deformation of the polyacrylamide‐based network and orientation of the Ad‐cellulose component.

### 2.7 Visualization of Real‐Time Mechano‐Responsive Behavior for Stress Sensing Applications

Simulation using finite element analysis was conducted to further explore the potential of the hydrogel as a stress‐sensing material. To evaluate the internal stress distribution under deformation, hydrogel samples were prepared with 5 mm circular and star‐shaped holes at the center, which enabled the stress concentration in specific regions to be visualized during stretching. When stretched, the gels exhibited clear whitening around the edges of the holes along the stretching direction, indicating localized stress concentration (Figure 4g). This visual observation was in good agreement with the simulation results, which revealed that the highest stress values were concentrated at the periphery of the hole, particularly along the major axis formed by the deformation. This optical response was quantified by analyzing the degree of whitening by measuring the mean gray value in the representative regions (A–C) of the hydrogel images. The mean gray value increased with the applied strain, with the highest values observed near the periphery of the hole (region A), where the stress concentration was most severe. In contrast, region C exhibited low turbidity, reflecting the relatively low stress experienced by this region. These trends were consistent across both the circular and star‐shaped geometries. The spatial distribution of the whitening behavior was also visualized by generating pseudocolor images by applying a lookup table (LUT) to convert the grayscale values of the optical images to colors (Figure S20). The color distribution patterns in the LUT images were in reasonable agreement with the stress distribution profiles predicted by the finite element simulations in Figure 4g, particularly around the stress concentration regions near the periphery of the holes. In addition, systematic changes in the color distribution of the LUT images were observed with increasing strain, indicating that the proposed method can sensitively visualize changes in the local mechanical loading during deformation. Although this approach does not provide exact quantitative stress values, it is a useful semiquantitative stress‐mapping method for visually capturing relative changes in the local mechanical loading and stress concentration behavior during deformation.

## 3 Conclusion

In this study, we demonstrated continuous and reversible stress visualization of hydrogels, enabled by supramolecularly switched stretch‐induced phase separation. Our design strategy involved the synthesis of Ad‐cellulose as a guest‐functionalized phase‐separation domain capable of on–off switching of hydration in response to host–guest complexation. The incorporation of these domains into the gel network enabled the gels to produce a clear optical contrast under visible light in response to the application of mechanical stimuli. The CD–AdC gels underwent reversible and repeatable transparency–opacity transitions during loading–unloading cycles, with the degree of turbidity corresponding approximately linearly to the applied stress. This optical response was maintained over repeated deformation cycles, and its behavior, which closely depended on the stress relaxation and strain rate, indicates that the turbidity changes reflect the internal stress state of the hydrogel network.

Structural analyses provided complementary insights into the mechano‐responsive behavior of the gels. NMR spectroscopic analyses provided evidence for the desolvation of Ad‐cellulose under stretching. SAXS and SANS analyses revealed that macroscopic stretching induces anisotropic deformation of the hydrogel network and the orientation of the Ad‐cellulose component. Furthermore, the successful extension of this design strategy to alternative polymer backbones, with Ad‐PVA serving as the mechanoresponsive domain, underscores the generality and modularity of the concept of supramolecular phase separation.

Importantly, the gels enable the direct visualization of internal stress distributions. These distributions were closely aligned with the results of the finite element simulation and highlighted their potential for real‐time stress mapping. Overall, this work establishes supramolecular switching mechanotransduction (SSM)—based on stretch‐induced phase separation enabled by a host–guest molecular switch—as a powerful and versatile mechanism for mechanoresponsive hydrogels and provides a general framework for converting molecular interactions into macroscopic, reversible, and semiquantitative mechanical readouts. This supramolecular switch‐based strategy establishes a versatile platform for real‐time, spatially resolved stress visualization in soft materials across a wide range of mechanical stimuli, opening new opportunities in soft robotics, wearable systems, biointerfaces, and mechanobiology.

## 4 Experimental

### 4.1 Materials

Cellulose powder Avicel PH‐101, lithium chloride (LiCl), ammonium persulfate (APS), (2‐hydroxypropyl)‐β‐cyclodextrin (HP‐β‐CD), and methyl‐β‐cyclodextrin (methyl‐β‐CD) were purchased from Sigma–Aldrich. *N, N*‐Dimethylacetamide (DMAc), *N*, *N*‐dimethylacrylamide (DMAAm), acrylamide (AAm), and pyridine were purchased from FUJIFILM Wako Pure Chemical Corporation. 1‐Adamantanecarbonyl chloride (AdCOCl), 1‐adamantanamine hydrochloride (Ad‐NH_3_Cl) and 2‐hydroxy‐4'‐(2‐hydroxyethoxy)‐2‐methylpropiophenone (Irgacure 2959) were purchased from Tokyo Chemical Industry Co., Ltd. Poly(vinyl alcohol) (PVA, DP ≈ 2000) was purchased from KISHIDA Chemical Co., Ltd. 6‐Acrylamido‐β‐cyclodextrin (β‐CDAAm) was provided by Yushiro Chemical Industry Co., Ltd. Acrylamide (2,3,3‐D_3_, 98%) (AAm‐d_3_) and deuterium oxide (D_2_O, 99.96%) were purchased from Cambridge Isotope Laboratories Inc. *N*‐Adamantyl acrylamide (Ad‐AAm) was synthesized according to a previously reported procedure. All reagents were used as purchased without further purification.

### 4.2 Preparation of Ad‐Cellulose

Ad‐cellulose was synthesized by esterification between the hydroxyl groups of cellulose and AdCOCl (Figure S1a). Cellulose, Avicel (6 g) was added to DMAc (300 mL) and stirred in an oil bath for 1 h at 150°C. The slurry was then cooled to 90°C, and LiCl (24 g) was added under stirring. After stirring for 1 h at room temperature, the cellulose was completely dissolved in the DMAc/LiCl solution. Pyridine (7.03 g, 2.4 eq. to the anhydro glucose unit (AGU)) was added to the cellulose solution, and the solution was heated at 60°C. AdCOCl (8.83 g, 1.2 eq. to AGU) was added and stirred continuously for 18 h. After the reaction, the solution was poured into 2 L of methanol, and the precipitate was filtered and re‐dissolved in DMSO, followed by precipitation and filtration. Then, the crude product of Ad‐cellulose was obtained after vacuum drying at 80°C. The dried sample (12 g) was added to an aqueous solution of HP‐β‐CD and stirred for 3 h at 80°C. The aqueous solution of HP‐β‐CD was prepared by dissolving HP‐β‐CD (24 g) into distilled water (40 mL). The polymer was dissolved in aqueous solutions of HP‐β‐CD and filtered under vacuum to remove insoluble particulates. The resulting solution was precipitated with methanol and filtered. After vacuum drying at room temperature, the product was dissolved in DMSO and precipitated from a methanol/water mixture (1/1 (v/v)) for further purification. After precipitation, the dispersion was exchanged with methanol, followed by water by centrifugation to remove residual HP‐β‐CD. Finally, Ad‐cellulose was obtained by lyophilization of the aqueous solution.

The degree of substitution (DS) of Ad‐cellulose was determined by ^1^H NMR spectroscopy (Figure S2). The DS was calculated as 0.2 using Equation (1). This calculation utilized the integral areas of Ad protons (I_(Ad)_) and the H1 proton of AGU (I_(AGU(H1))_).

(1) $$\mathrm{DS}=\left(I_{\left(\mathrm{Ad}\right)}\;\times 1\right)/\left(I_{\left(\mathrm{AGU}(H1)\right)}\;\times 15\right)$$

### 4.3 Preparation of Mechano‐Responsive Supramolecular Hydrogels (CD−AdC*x* Gel)

CD−AdC*x* hydrogels with different concentrations of Ad‐cellulose were prepared by free‐radical polymerization of AAm and β‐CDAAm in the presence of Ad‐cellulose, where *x* refers to the weight percentage of Ad‐cellulose in the pre‐gel solution. Ad‐cellulose contents of 8, 10, and 12 wt.% and β‐CDAAm at feed amounts of 0.12, 0.15, and 0.18 mmol were dissolved in water, respectively. The concentration of the AAm monomer was fixed at 2.0 mmol/g(water). APS (2.0 × 10^−2^ mmol/g(water)) was added as an initiator. After degassing and centrifugation, the mixture was poured into a mold, and polymerization was performed at 50°C for 3 h. The resulting hydrogels were immersed in water for three days to remove residual impurities. The preparation procedure for CD−AdC8_AAmd_3_ was identical to that for CD−AdC8 gel, except that AAm‐d_3_ was used instead of AAm.

### 4.4 Preparation of Hydrogels with Competitive Molecules

For control experiments, CD‐AdC8 hydrogels were immersed in 10 mm aqueous solutions of the guest or host competitive molecules, Ad‐NH_3_Cl and methyl‐β‐CD, respectively, for 24 h.

### 4.5 Preparation of AdC_HG Hydrogels

The AdC_HG hydrogels were prepared following the same polymerization procedure as described for the CD–AdC8 gels, except that AAm was replaced with DMAAm, and the host–guest (HG) cross‐linker (5 or 10 mol% relative to DMAAm) was added to the precursor solution. All other polymerization and post‐treatment conditions were identical to those described above. The HG cross‐linker was prepared by dissolving Ad‐AAm and β‐CDAAm in water at 80°C for 2 h at a monomer molar ratio of 1:1, followed by filtration and lyophilization after complete dissolution.

### 4.6 Preparation of Ad‐PVA

Ad‐PVA was synthesized via esterification between the hydroxyl groups of PVA and AdCOCl. PVA (5.0 g) was added to DMAc (95 g) and stirred in an oil bath at 90°C. Subsequently, LiCl (5.0 g) was added under stirring. After stirring for 1.5 h at room temperature, the PVA was completely dissolved in the DMAc/LiCl solution. Pyridine (7.1 g, 0.8 eq. to the hydroxy group of PVA) was added to the PVA solution, and the solution was heated at 60°C. AdCOCl (8.9 g, 0.4 eq. with respect to the hydroxyl group of PVA) was added and stirred continuously for 17 h. After the reaction, the solution was poured into distilled water (2.5 L), and the precipitate was filtered and redissolved in DMF. The crude Ad‐PVA product was obtained by precipitation with acetone (2.5 L) and filtration. The obtained crude Ad‐PVA product was added to distilled water (120 g), and the residual acetone and water were removed by evaporation. The Ad‐PVA was added to distilled water (100 g), and HP‐β‐CD (25 g) was added to the mixture. The mixture was stirred for 15 min at 90°C. HP‐β‐CD (13 g) was further added, and the mixture was stirred for 1 h at 90°C. Ad‐PVA was dissolved in aqueous solutions of HP‐β‐CD and vacuum filtrated to remove insoluble Ad‐PVA. The collected solution was precipitated with 3.5 L of a water/acetone mixture (6/1, (v/v)) and filtered. Ad‐PVA was dissolved in DMF by evaporating water and acetone, and then precipitated in a water/acetone mixture (4/1 (v/v)) for further purification. After precipitation, the Ad‐PVA was collected using a stainless‐steel test sieve. The obtained Ad‐PVA was added to distilled water, and the residual acetone was removed by evaporation. Ad‐PVA was obtained after lyophilization of the mixture.

### 4.7 Preparation of Ad‐PVA Hydrogels

The Ad‐PVA hydrogels were prepared by free‐radical polymerization of DMAAm and β‐CDAAm in the presence of Ad‐cellulose. Ad‐PVA (10 wt.%) and β‐CDAAm (100 mol%; 1.0 eq to the Ad group of Ad‐PVA) were dissolved in water. The concentration of the DMAAm monomer was 2.0 mmol/g (water). Irgacure2959 (0.01eq. to DMAAm) was added as the photoinitiator. After degassing and centrifugation, the mixture was poured into a glass and molded, followed by UV polymerization for 30 min. The hydrogels were immersed in water for three days to remove unreacted monomer.

### 4.8 Preparation of PAAmd3‐HG Hydrogel

The PAAmd3‐HG hydrogel was prepared by free‐radical polymerization of AAm‐d3, Ad‐AAm, and β‐CDAAm. HG (6.0 molar ratio to AAm) was used as the cross‐linker. The preparation procedure is the same as for CD‐AdC8 gel. The HG cross‐linker was prepared by dissolving Ad‐AAm and β‐CD‐AAm in water at 80°C for 2 h at a monomer molar ratio of 1:1, followed by filtration and lyophilization after complete dissolution.

### 4.9 Characterization of Ad‐Cellulose

Attenuated total reflection Fourier transform infrared (ATR‐FTIR) spectra (Thermo Scientific Nicolet iS 5, USA) were recorded to confirm the successful preparation of the Ad‐cellulose. Details of the characterization are included as Supporting Information.^1^H NMR spectra were acquired to calculate the degree of substitution of the Ad group in the cellulose derivatives. ^1^H NMR spectra were recorded in DMSO‐d6 using a JNM‐ECS400 spectrometer (400 MHz; Jeol, Japan).

### 4.10 Measurement of Mechanical Properties of Hydrogels

Tensile tests on the hydrogels were performed using a universal tensile machine (Shimadzu EZ Graph, Japan) at a velocity of 30 mm/min. The toughness of the hydrogels was calculated by integrating the area under the stress–strain tensile curves. The Young's modulus of the hydrogels was calculated as the slope of the initial linear region of the stress–strain tensile curves. For the recoverability test, a time‐dependent loading‐unloading tensile test was conducted up to 600% strain at a speed of 50 mm/min. Hydrogels with uniform sizes of 5–10 mm were used in the tests.

### 4.11 Evaluation of Mechano‐Responsive Properties of Hydrogels

To evaluate the mechanoresponsive properties, loading‐unloading tensile tests were carried out up to 600% strain at a speed of 50 mm/min. Additionally, repetitive loading‐unloading tensile tests were performed on the ring‐shaped hydrogels for 20 cycles up to 400% strain at a speed of 500 mm/min in air. For the stress–relaxation test, a loading speed of 40 mm/min was applied until a strain of 400% was reached, after which the strain was maintained for 4 min. The stresses during the loading and relaxation were also recorded. To investigate the tensile speed‐dependent mechanoresponsive behavior, tensile tests were conducted at speeds of 0.5, 5, and 500 mm/min. Hydrogels with uniform sizes of 5–10 mm were used in the tests. Stress relaxation and tensile speed‐dependent mechano‐responsive behavior tests were performed in water. The mean gray values of the sample images were calculated using ImageJ by dividing the sum of the gray values by the total number of gray values within the selected region. The region of interest was specifically chosen from the central area of the hydrogels to ensure a representative calculation of the mean gray value. To visualize the turbidity distribution during deformation, pseudocolor images were generated by applying a lookup table (LUT) to the grayscale optical images using the ImageJ software.

### 4.12 Small‐Angle X‐ray Scattering (SAXS) Measurements

The SAXS analysis was performed using a BL40B2 diffractometer installed at SPring‐8 (Nishiharima, Japan). A synchrotron beam wavelength (*λ*) of 1.2 Å was used during the SAXS measurements. A camera length of 2.27 m was used for SAXS, and scattering was recorded using a PILATUS 2 m detector (Decris). An extension device (Linkam 10073 L) was installed in the tensile direction of the hydrogel and set perpendicular to the X‐ray beam in beamline BL40B2. SAXS images were continuously recorded during tensile testing.

### 4.13 Small‐Angle Neutron Scattering (SANS) Measurements

SANS was performed using a SANS‐J diffractometer installed at the Japan Research Reactor‐3 (JRR‐3) of the Japan Atomic Energy Agency (JAEA) in Tokai, Japan. The hydrogels were soaked in D_2_O, and the solvent was replaced three times to ensure D_2_O exchange. To compensate for the decrease in the sample thickness upon stretching, four gels were stacked at 400% strain to ensure sufficient scattering intensity. The incident neutron beam was monochromatized using a velocity selector to obtain a wavelength (*λ*) of 0.65 nm and a wavelength distribution (Δ*λ*/*λ*) of 0.15. A 20 mm × 20 mm aperture 10 m upstream from the sample and a 14 mm‐diameter aperture at the sample position defined the size of the incident neutron beam. Two 2D position‐sensitive detectors were used to detect the neutrons scattered from the sample. One was a 650 mm × 680 mm (height × width) detector comprising 95^3^He‐tube detectors (8 mm in diameter), and the other was a 650 mm × 385 mm (height × width) detector consisting of 48^3^He‐tube detectors (8 mm in diameter). The sample‐to‐detector distances were 2, 4, and 10 m along the beam path of the incident neutrons, whereas that of the smaller detector was 0.96 m. Additionally, a *q* region of ultra‐small angle neutron scattering (0.01< *q* < 5 nm^−1^), was measured by using a set of neutron lenses and a position‐sensitive photomultiplier. This instrument configuration covered the *q* region of 0.001 < *q* < 10 nm^−1^. The 2D scattering data recorded by these three detectors were corrected using the detection efficiency before azimuthal averaging was applied to obtain the scattering intensity distribution as a function of *q*. The instrument background and air scattering data were subtracted from each scattering profile. Absolute scattering intensity as a function of *q*(= (4π/*λ*)sin*θ*), *I*(*q*) (cm^−1^), was obtained using an Al plate as a standard reference. *λ* and 2*θ* are the wavelength of the incident neutrons and the scattering angle, respectively. Quartz cell scattering was subtracted from the sample scattering by considering the transmittance of neutrons in each sample. Solvent scattering and incoherent solute scattering were subtracted from the sample scattering, considering the solvent and solute volume fractions, respectively, calculated from the sample composition, where solute incoherent scattering was estimated from the chemical composition of the solute.

### 4.14 NMR Measurement of the Hydrogels

To investigate the change in the solubility of Ad‐cellulose within the gel network upon mechanical deformation, ^1^H NMR measurements were performed on the CD−AdC_AAmd_3_ hydrogel in both its unstretched state and when stretched to 300% strain. Prior to the measurement, the hydrogel samples were thoroughly solvent‐exchanged from H_2_O to D_2_O. In the stretched state, the gel was elongated to 300% strain and fixed in the stretched configuration by using a thread to secure both ends to a rigid rod. The rod‐supported sample was then carefully inserted into an NMR tube, allowing the gel to maintain the applied strain during the measurement. All spectra were recorded in D_2_O, and changes in the signal intensity of the Ad‐cellulose‐related peaks upon stretching were analyzed.

### 4.15 Finite Element Analysis

Finite element analysis (FEA) was performed using the Code Aster software to evaluate the stress distribution of the hydrogel during uniaxial tensile deformation. The hydrogel specimen was modeled as a rectangular shape with dimensions of 10 mm × 10 mm × 1 mm, containing a central through‐hole with a diameter of 5 mm. Two different hole geometries, circular and star‐shaped, were introduced at the center, and simulations were performed for each model. A 3D Neo‐Hookean hyperelastic material model was adopted for the hydrogel with parameters C_10_ = 8.33 and Poisson's ratio ν = 0.49. A nonlinear static analysis was performed using Green–Lagrange deformation measurements. The resulting stress distribution was evaluated using the nodal von Mises equivalent stress (SIEQ_NOEU) obtained from the finite element simulations, which were visualized to identify the stress concentration and spatial stress heterogeneity induced by different hole geometries.

## Author Contributions

A.S. conceived the study. H.U. and Y.T. reviewed the manuscript and provided critical suggestions for revisions. S.N. and N.I. conducted the experiments. S.N. and A.S. wrote the manuscript. T.K., Y.U., and R.M. performed the structural analysis based on the SAXS and SANS measurements and contributed to the interpretation of the results. All the authors discussed the results and approved the final version of the manuscript.

## Conflicts of Interest

The authors declare no conflicts of interest.

## Supporting information




**Supporting File 1**: advs76110‐sup‐0001‐SuppMat.docx.

**Supporting File 2**: advs76110‐sup‐0002‐MovieS1.mov.

**Supporting File 3**: advs76110‐sup‐0003‐MovieS2.mov.

**Supporting File 4**: advs76110‐sup‐0004‐MovieS3.mov.

## Data Availability

The data that support the findings of this study are available from the corresponding author upon reasonable request.
