# Supplementary material for: Real‐Time Stress Visualization of Hydrogels Enabled by Supramolecularly Switched Stretch‐Induced Phase Separation
Source: Adv Sci (Weinh). 2026 Jun 18;13(51):e76110. doi: 10.1002/advs.76110 (PMC13336535; doi:10.1002/advs.76110)
Supplement: Supplementary file 1 [file ADVS-13-e76110-s001.docx]

Supplementary Information

**Real-time stress visualization of hydrogels enabled by supramolecularly switched stretch-induced phase separation**

Sooyeon Noh^1^, Akihide Sugawara^1^*, Naoaki Ishihara^1^, Takashi Konishi^2^, Yuki Ueda^3^,

Ryuhei Motokawa^3^, Yoshinori Takashima^4^ and Hiroshi Uyama^1^*

^1^Department of Applied Chemistry, Graduate School of Engineering, The University of Osaka, 2-1 Yamadaoka, Suita, Osaka 565-0871, Japan

^2^ Graduate School of Human and Environmental Studies, Kyoto University, Yoshida-nihonmatsu-cho, Sakyo-ku, Kyoto 606-8501, Japan

^3^ Materials Sciences Research Center, Japan Atomic Energy Agency, Tokai, Ibaraki 319-1195, Japan

^4^ Department of Macromolecular Science, Graduate School of Science, Institute for Open and Transdisciplinary Research Initiatives (OTRI), Forefront Research Center, The University of Osaka, 1-1 Machikaneyama-cho, Toyonaka, Osaka 560-0043, Japan

Phone: +81-6-6879-7364

Fax: +81-6-6879-7367

AS: a_sugawara@chem.eng.osaka-u.ac.jp

HU: uyama@chem.eng.osaka-u.ac.jp

**Figure S1.** (a) Preparation of Ad-cellulose. (b) FTIR spectra of Ad-cellulose, cellulose, and AdCOCl.

**Characterization of Ad-cellulose**

The successful modification of cellulose with Ad groups was verified using FTIR spectroscopy. As shown in **Figure S1(b)**, the FTIR spectra of Ad-cellulose displayed characteristic absorption bands at 1700 cm^−1^ and 2900 cm^−1^, corresponding to the ester carbonyl C=O stretching and CH stretching vibrations of the incorporated Ad unit, respectively. The broad band at 3000 cm^−1^, attributed to the OH stretching vibrations, confirmed the presence of unmodified hydroxyl groups. These results indicated that the Ad groups were effectively grafted onto the cellulose backbone via esterification.

**Figure S2.** ^1^H NMR spectrum of Ad cellulose in DMSO-d6.

**Figure S3.** ^1^H NMR spectra of Ad-cellulose, β-CDAAm, and Ad-cellulose and β-CDAAm in D_2_O.

**Figure S4.** FTIR spectra of AAm, β-CDAAm, and CD–AdC8 gel.

**Figure S5.** ^1^H NMR spectra of CD–AdC8 precursor solution before and after polymerization.

**^1^H NMR (D_2_O) of CD-AdC8 before polymerization):** δ = 5.5–6.0 ppm (vinyl protons of AAm and β-CDAAm), 4.8–3.5 ppm (β-CD protons and overlapping signals from AAm and β-CDAAm), 1.6–2.1 ppm (Ad protons of Ad-cellulose), 6.9 & 7.5 ppm (NH_2_ of acrylamide).

**Figure S6.** Photographs of CD–AdC8 and CD–AdC10 gels during the loading-unloading cycle test.

**Figure S7.** Stress–strain curves of CD–AdC gels during loading-unloading cycle tests.

**Figure S8.** Stress–strain curve of ring-shaped CD–AdC8 gel during a loading-unloading cycle test.

**Figure S9.** (a) Photographs of CD–AdC8 gel during repetitive cyclic testing. (b) Stress–strain curves of CD–AdC8 gel recorded during repeated cyclic tests.

**Figure S10.** Photographs of CD–AdC8, CD–AdC10, and CD–AdC12 gels during the stress–relaxation test.

**Figure S11**. Non-normalized mean gray values of CD–AdC gels during the stress relaxation test.

**Figure S12.** Photographs of CD–AdC8 gel during tensile tests at tensile speeds of 500, 50 and 0.5 mm/min.

**Figure S13.** Stress–strain curves of CD–AdC8 gel at different tensile speeds.

**Figure S14.** Stress–strain curves of CD–AdC8 gels immersed in aqueous solutions of the competitive guest (Ad-NH_3_Cl) and host (methyl-β-CD).

**Figure S15.** Photographs of AdC_HG0, AdC_HG5, and AdC_HG10 gels during the loading–unloading cycle test.

**Figure S16.** Photographs of the CD–AdC8 gel stretched to 300% strain, affixed to a rigid rod using a thread, and inserted into an NMR tube for ¹H NMR measurement.

**Figure S17.** ^1^H NMR spectra of PAAmd3-HG gels in D_2_O with and without 300% stretching.

**Figure S18.** SEM images of lyophilized CD–AdC8 gel after tensile testing.

**Figure S19.** Schematic energy landscape illustrating the reversible formation of heterogeneous states in a hydrogel network under mechanical stretching. (A) In the absence of stress, the hydrated homogeneous state is energetically favored, whereas the locally dehydrated heterogeneous state exists as a metastable higher-energy state. (B) Under stretching, the energy landscape is altered such that the heterogeneous state becomes relatively stable, leading to stress-induced local dehydration and optical heterogeneity. Upon unloading or relaxation, the system returns to a hydrated homogeneous state, which is consistent with the experimentally observed reversible transparency change.

**Figure S20.** Pseudocolor LUT images generated from optical photographs of CD–AdC hydrogels with (a) circular and (b) star-shaped holes under tensile deformation (initial chuck length = 1 cm).
